# A Systematic Review and Meta-analysis on Innovative Approaches in Tuberculosis Diagnosis: Challenges and Future Directions

**DOI:** 10.21106/IJMA_29_2025

**Published:** 2026-06-13

**Authors:** Ahmad Abdulhadi, Nasiru Abdullahi, Ibrahim Yusuf, Basheer I. Waziri, Kasimu Mamuda, Husna F. Ibrahim, Khadija Muhammad, Maryam M. Ibrahim, Aisha A. Abdullahi, Hassan A. Murtala, Muhammad A. Abbas, Hamisu M. Salihu

**Affiliations:** 1 Department of Microbiology, Division of Tuberculosis and Antimicrobial Resistance Research, Kano Independent Research Center Trust, Nigeria; 2 Department of Genomics and Molecular Biology Research, Kano Independent Research Center Trust, Nigeria; 3 Department of Biochemistry, College of Basic Medical Science, Bayero University Kano, Nigeria; 4 Department of Microbiology, College of Natural and Pharmaceutical Science, Bayero University Kano, Nigeria; 5 Department of Physiology, College of Basic Medical Science, Bayero University Kano, Nigeria; 6 National Tuberculosis Reference Laboratory, NTBLTC Saye, Zaria, Kaduna, Nigeria; 7 Department of Epidemiology and Population Health, Kano Independent Research Center Trust, Nigeria; 8 Kano Center for Disease Control and Prevention, Nigeria; 9 Department of Community Medicine, College of Health Sciences, Bayero University Kano, Nigeria

**Keywords:** Diagnosis, Innovative Approaches, Review, Screening, Tuberculosis

## Abstract

**Background and Objective:**

Tuberculosis (TB) has continued to be one of the global threats, affecting millions of individuals globally, including the maternal and child health (MCH) populations and individuals with HIV/AIDS. TB infected 10.8 million individuals, leading to 1.25 million deaths across the globe annually, leaving 4 million missing cases contributing to the global TB burden. This study aims to unveil innovative approaches to TB diagnosis, challenges, and the future direction of this field.

**Methods:**

A comprehensive literature search was conducted to retrieve studies published between 2019 and 2024 from PubMed, Google Scholar, and Web of Science. The studies were reported following the Preferred Reporting Items for Systematic Reviews and Meta-Analyses guidelines and screened using the Rayyan tool. The quality and risk of bias of the included studies were assessed using Quality Assessment of Diagnostic Accuracy Studies 2 for diagnostic accuracy studies and Strengthening the Reporting of Observational Studies in Epidemiology for observational studies. A random effects model was employed to calculate the pooled sensitivity and specificity, while Egger’s test and funnel plots were utilized to evaluate publication bias. R and MetaDTA software were used for all the statistical analyses.

**Results:**

The review included 25 studies with sample sizes ranging from 100 to 6,520 participants and assessed various innovative approaches for diagnosing TB, including molecular methods, biomarker-based techniques, and artificial intelligence (AI) applications. The most common approach was molecular testing, specifically cartridge-based tests. The overall sensitivity of these methods was 0.79 (95% confidence interval [CI]: 0.70–0.86), with a heterogeneity index (I²) of 92.9% and a *p* < 0.0001. The specificity was recorded at 0.93 (95% CI: 0.87–0.96), with an I^2^ of 94.8% and a *p* < 0.0001.

**Conclusion and Global Health Implications:**

This review reinforces the promise of innovative diagnostic methods, especially cartridge-based molecular tests, for improving TB detection. However, moderate sensitivity and high heterogeneity emphasize the need for cautious implementation and further validation of new tools such as the AI-based and biomarker-based. Strategic investment in research and contextual deployment is critical for closing the TB diagnosis gap globally.

## INTRODUCTION

For more than a century, tuberculosis (TB) has remained a formidable disease, with 10.8 million new cases and 1.25 million deaths reported in 2024.^[1]^ Although it is a preventable and curable disease with high rates of treatment success when diagnosed and treated early, it remains the biggest threat to public health, mostly in resource-limited countries, where fully functional diagnostic laboratories are scarcely available.^[2]^ Approximately 70% of low- and middle-income countries (LMICs) have laboratories.^[3]^ Acid-fast bacilli (AFB) smear microscopy is a technique used to detect *Mycobacterium* in sputum samples collected from suspected patients.^[4]^ Although it is rapid and cost-effective for TB screening, it has a low sensitivity of approximately 40%,^[5]^ making it unsuitable for TB diagnosis. In addition, the TB culture technique, which involves isolating *Mycobacterium tuberculosis* (MTB) from pulmonary or extrapulmonary specimens, is considered the gold standard for TB confirmation, but it is time-consuming (6–8 weeks), which affects the timely confirmation of TB, leading to delayed intervention.^[6,7]^ To achieve the World Health Organization’s (WHO) strategic plan to stop TB by 2030, there is a pressing need for an alternative, rapid, sensitive, and accessible approach to diagnose TB.

To solve the above problem, many innovative technologies that employ techniques such as molecular methods, biomarkers, and artificial intelligence (AI) have been developed to increase TB diagnosis in terms of sensitivity, specificity, turnaround time, and detection of resistance to first- and second-line anti-TB drugs.^[3]^

In the past decade, GeneXpert has emerged as an innovative solution for TB diagnosis, particularly in LMICs, such as India. This technique provides rapid detection of TB and rifampicin resistance within 2 h,^[8]^ significantly accelerating diagnosis and treatment initiation. However, despite its diagnostic speed and accuracy, the system remains expensive to acquire and sustain, especially in settings where the TB burden is high and healthcare-seeking behavior is low.^[9]^ In many LMICs, the deployment and maintenance of GeneXpert machines, along with the procurement of necessary consumables, have largely relied on funding from global health initiatives and international partners.^[9]^ Reliance on external financing may not be a viable long-term solution, emphasizing the need for sustainable and locally adapted diagnostic strategies.

Although systematic reviews have explored various TB diagnostic tools and innovative approaches, a notable gap persists in synthesizing the intersection of innovation, affordability, self-sustainability, and potential for community-based screening. This systematic review aims to address this gap by exploring the following question: (i) To what extent are emerging innovative approaches for TB diagnosis improving affordability, turnaround time, sensitivity, and the feasibility of community-based screening to enhance TB detection? (ii) What promise do these innovations hold for achieving sustainable diagnostic solutions?

This review catalogs emerging diagnostic tools, evaluates their global acceptance, assesses their ability to overcome existing challenges, and provides insights into future directions for advancing TB diagnostics on a sustainable scale.

## METHODS

### Literature Search

This study was conducted according to the Preferred Reporting Items for Systematic Reviews and Meta-Analyses (PRISMA). A comprehensive literature search was conducted across databases to identify articles relevant to innovative TB diagnostics. The following databases were searched: PubMed, Web of Science, and Publish or Perish (Google Scholar). The keywords used were “Innovative approaches” AND “Tuberculosis diagnosis” OR “Tuberculosis detection.” Along with the MeSH terms Humans; TB; TB, Pulmonary, Extrapulmonary. This search strategy was developed to capture the most up-to-date approaches to TB diagnosis. A 5-year limit was set to select the most recent studies, and English was used as the preferred language of publication. This review has been registered with Prospective Register of Systematic Reviews Online (PROSPERO) to prevent duplication, and it has been assigned the registration number CRD420251086597.

### Study Selection

After a comprehensive literature search, the retrieved studies were screened by two independent reviewers, A. A. and H.F.I., based on their titles and abstracts. Subsequently, full-text screening was performed to systematically identify relevant articles. The RAYYAN tool (https://rayyan.qcri.org/) was used with the blinding feature enabled to minimize bias. Any disagreements or conflicts between the reviewers during screening were resolved by inviting a third reviewer.

### Inclusion Criteria

The objective was to incorporate current studies highlighting any novel or innovative approaches for diagnosing TB, including peer-reviewed studies written in English and including human subjects. The inclusion criteria for selecting published articles were studies, reviews, or articles that reported (1) proposed improvements, predictive models, or scalable solutions for TB diagnostics. (2) Use molecular diagnostics (e.g., PCR-based methods), immunological assays, AI-based interpretations, or biomarker-based approaches. (3) within the past 5 years, and (4) analyze barriers such as cost, accessibility, accuracy, implementation, or societal factors affecting innovative diagnostic adoption.

### Exclusion Criteria

The following exclusion criteria were implemented to uphold the relevance and integrity of this research: (1) Studies or articles discussing traditional methods (e.g., sputum smear microscopy) without reference to innovation or improvements; (2) research focused solely on TB treatment, epidemiology, or prevention without relevance to diagnosis or non-human research; (3) reviews or summaries that do not add new insights or duplicate findings from previously included works; (4) content focusing on diagnostic innovations for diseases other than TB; and (5) discussions unrelated to diagnostic challenges, such as policy, funding, or unrelated healthcare system issues. Studies published in languages other than English were also excluded.

### Data Extraction

The data components for this systematic review included the author and publication year, study design, sample size, country of study, diagnostic method assessed, principal findings, and identified limitations.

### Quality and the Risk of Bias Assessment

The quality and risk of bias assessments for the included studies were conducted by two reviewers, A. A and H.F.I. using specific guidelines: Quality Assessment of Diagnostic Accuracy Studies 2 (QUADAS-2) for diagnostic accuracy studies and Strengthening the Reporting of Observational Studies in Epidemiology (STROBE) for observational studies

### Data Synthesis

Data extracted from each of the selected studies included the author(s) and year, sample size, country, study design, diagnostic method evaluated, key findings, sensitivity, and specificity. The data were analyzed based on the innovative approaches assessed and reported, ranging from molecular, biomarker-based, and AI-based. And then, a meta-analysis was conducted to provide a comprehensive synthesis of the review.

### Statistical Analysis

Statistical analysis was conducted using the R programming language version 4.5.1, along with the meta and meta for packages, as well as the meta DTA software. Data were pooled using a random-effects model, and the I² statistic, which measures inconsistency, was employed to evaluate heterogeneity across studies. I^2^ values of 25%, 50%, and 75% indicate low, moderate, and high heterogeneity, respectively. In addition, Egger’s test was utilized to assess publication bias, and a funnel plot was created to visualize this assessment.

## RESULTS

**Figure 1:**
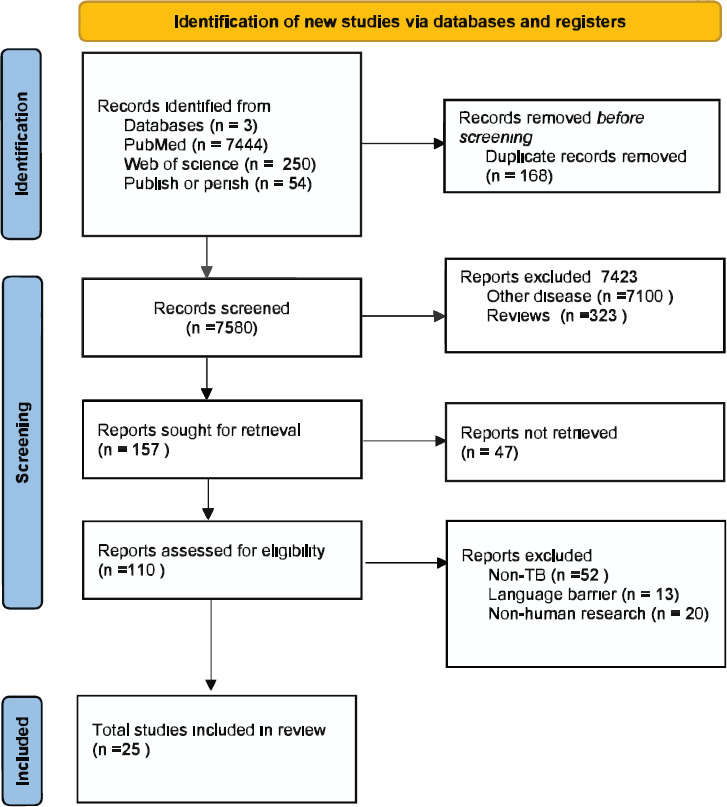
PRISMA framework showing the inclusion of studies in this systematic review.

A total of 7,748 articles were retrieved from PubMed (7,444), Publish or Perish (Google Scholar) (54), and Web of Science (250). After removing 168 duplicates, 7,580 unique articles were screened based on their title and abstracts. Following this screening process, 7,423 articles were excluded, and a full-text screening was conducted on 157 articles. Ultimately, 110 full-text articles were assessed, of which 25 studies met the inclusion criteria and were included in the final analysis [Figure 1].^[10]^

A comprehensive summary of the included studies is presented in Table 1. Key details, such as the author, study design, country, sample type, diagnostic method evaluated, key findings, and limitations, are highlighted. Among the 25 studies analyzed, 15 focused on molecular diagnostic approaches,^[6,11-24]^ five on biomarker-based approaches,^[25-29]^ three on AI-based approaches,^[30-32]^ one on an electronic nose,^[33]^ and one on an immunochromatographic strip.^[34]^

**Table 1:** Summary of the diagnostic approach evaluated (*n*=25).

Author and References	Sample size	Country	Study design	Diagnostic method	Key findings	Sens	Spec
Chen et al., 2024^[11]^	1000	China	DA	AI Microscopy	TB screening	91.94	97.97
John et al., 2023^[12]^	5297	Nigeria	DA	AI CXR	TB screening	89.4	62.8
Moore et al., 2023^[13]^	100	South Africa	DA	CAPTURE-XT	TB diagnosis	87	100
De vos et al., 2021^[14]^	1698	South Africa	DA	Cobas MTB	TB diagnosis	92	100
Liu et al., 2023^[15]^	1492	China	DA	Melt pro TB	TB diagnosis	69	97.1
Sossen et al., 2024^[16]^	1731	Malawi	DA	Urine Xpert Ultra	TB diagnosis	32.7	98
Ko and Yoon, 2024^[6]^	114	Korea	DA	BD MAX MDR TB	PTB diagnosis	79.4	88.8
Shi et al., 2021^[17]^	5946	China	DA	Genechip	TB Diagnosis	70	99
Huerga et al., 2023^[18]^	1575	Uganda	DA	FujiLAM	POC	60	87
Phunphae et al., 2024^[19]^	129	Thailand	DA	IC strip	TB diagnosis	93.9	93
Song et al., 2021^[20]^	103	China	DA	TB LAMP	TB diagnosis	82.6	94.5
Muyoyeta et al., 2021^[21]^	151	Zambia	DA	FujiLAM	TB detection	77	92
Dippenaar et al., 2021^[22]^	610	South Africa	DA	FlouroType MTB	TB diagnostic	91.6	93.8
Li et al., 2022^[23]^	108	China	DA	MALDI-TOF	TB diagnosis	72.7	100
Broger et al., 2020^[24]^	372	Peru	DA	FujiLAM	TB diagnosis	53.2	98.9
Abdulgader et al., 2024^[25]^	498	South Africa	DA	Truenaat MTB	TB diagnosis	84	95
Gao et al., 2024^[26]^	150	China	DA	NGS	TB diagnosis	88.1	94.1
Sawatpanich et al., 2022^[27]^	6520	Thailand	DA	Anyplex MTB	TB diagnosis	79.7	94.5
Fu et al., 2022^[28]^	5390	Taiwan	RS	AI Microscopy	TB screening	85.7	96.9
Yuan et al., 2024^[29]^	2620	China	CS	SIRI/CMI	MDR screening	86.1	82.1
Kagujje et al., 2023^[30]^	784	Zambia	CS	CRP	TB screening	86.4	34.8
Ule Belotti et al., 2022^[31]^	1575	Brazil	CS	GeneXpert	TB diagnosis	94.55	95.97
Coronel Texeira et al., 2021^[32]^	160	Paraguay	CS	Electronic nose	TB screening	N/A	N/A
Garba et al., 2023^[33]^	1454	Nigeria	RS	GeneXpert	TB diagnostic	N/A	N/A
Liu et al., 2024^[34]^	273	China	CS	NanoTNGS	TB diagnosis	N/A	N/A

TBL: Tuberculosis, MTB: *Mycobacterium tuberculosis*, NGS: Next-generation sequencing, TNGS: Targeted next-generation sequencing, SIRI: Systemic inflammation response index, CMI: Cardiometabolic index, LAM: Lipoarabinomannan, DA: Diagnostic accuracy, CS: Cross-sectional, RS: Retrospective, Sens: Sensitivity, Spec: Specificity, N/A: Not Available

The quality and risk of bias of the included studies were evaluated using the Quality Assessment of Diagnostic Accuracy Studies 2 (QUADAS-2) for diagnostic accuracy studies^[6,14-30]^ and the STROBE for observational studies [Tables 2 and 3].^[24-28,35]^

**Table 2:** Summary of the quality assessment and the risk of bias using the QUADAS-2 tool, *n* = (18).

Study	Risk of bias				Applicability concern		
	Patient selection	Index test	Reference standard	Flow and timing	Patient selection	Index test	Reference standard
Chen et al., 2024^[30]^	Low	Low	Low	Unclear	Low	Low	Low
John et al., 2023^[31]^	Low	Unclear	Low	Low	Low	Low	Low
Moore et al., 2023^[11]^	Low	Low	Low	Low	Low	Low	Unclear
De vos et al., 202^[12]^	Low	Low	Low	Unclear	Low	Low	Unclear
Liu et al., 2023^[13]^	Low	Low	Low	Low	Low	Low	Low
Sossen et al., 2024^[14]^	Low	Low	Low	Unclear	Low	Low	Unclear
Ko and Yoon 2024^[6]^	Low	Low	Unclear	Low	Low	Low	Low
Shi et al., 2021^[15]^	Low	Low	Low	Unclear	Low	Low	Unclear
Huerga et al., 2023^[25]^	Low	Low	Low	Low	Unclear	Low	Low
Phunphae et al., 2024^[34]^	Unclear	Low	Low	Low	Unclear	Low	Unclear
Song et al., 2021^[16]^	Low	Low	Low	Low	Low	Low	Low
Muyoyeta et al., 2021^[20]^	Low	Low	Low	Low	Low	Low	Unclear
Dippenaar et al., 2021^[17]^	High	Low	Unclear	Low	Unclear	Low	Low
Li et al., 2022^[18]^	Low	low	High	Low	Low	Low	Unclear
Broger et al., 2020^[27]^	Low	Low	Low	Unclear	Low	Low	Low
Abdulqader et al., 2024^[19]^	Low	Low	Low	Unclear	Low	Low	Unclear
Gao et al., 2024^[20]^	Low	Low	Low	Low	Low	Low	Low
Sawatpanich et al., 2022^[21]^	Low	Low	Low	Low	Low	Low	Unclear

QUADAS-2: Quality Assessment of Diagnostic Accuracy Studies 2

**Table 3:** Summary of the quality assessment and the risk bias of the included studies using the STROBE tool, *n* = (7).

Studies	Title and Abstract	Backgrounds	Objectives	Study Design	Setting	Participants	Variables	Data measurement
Coronel Teixeira et al., 2021^[32]^	1	1	1	1	1	1	1	0
Fu et al., 2022^[36]^	1	1	1	1	1	1	1	1
Yuan et al., 2024	1	1	1	1	1	1	1	1
Kagujje et al., 2023^[30]^	1	1	1	1	1	1	1	1
Garba et al., 2023^[33]^	1	1	1	1	1	1	1	1
Ule belotte et al., 2022^[31]^	1	1	1	1	1	1	1	1
Liu et al., 2024^[34]^	1	1	1	1	1	1	1	1
**Studies**	**Bias**	**Study size**	**Quantitative variables**	**Statistical methods**	**Results (Participants)**	**Descriptive data**	**Outcome data**	**Main results**
Coronel Teixeira et al., 2021^[32]^	0	1	0	1	1	1	1	0
Fu et al., 2022^[36]^	1	1	1	1	0	0	0	1
Yuan et al., 2024^[29]^	0	1	1	0	1	1	1	1
Kagujje et al., 2023^[30]^	0	1	1	1	1	1	1	1
Garba et al., 2023^[33]^	0	1	1	1	1	1	1	1
Ule belotte et al., 2022^[31]^	1	1	1	1	1	0	1	1
Liu et al., 2024^[34]^	1	1	1	1	1	1	1	1
**Studies**	**Other analyses**	**Key results**	**Limitations**	**Interpretation**	**Generalizability**	**Funding**	**Overall score**	
Coronel Teixeira et al., 2021^[32]^	1	1	1	1	1	0	70	
Fu et al., 2022^[36]^	0	1	0	1	1	1	75	
Yuan et al., 2024^[29]^	1	1	1	1	0	0	82	
Kagujje et al., 2023^[30]^	1	1	1	1	0	1	91	
Garba et al., 2023^[33]^	0	1	1	1	1	1	91	
Ule belotte et al., 2022^[31]^	0	1	1	1	0	1	86	
Liu et al., 2024^[34]^	0	1	1	1	1	1	95	

1: Reported, 0: Not reported, STROBE: Strengthening the Reporting of Observational Studies in Epidemiology

This structured approach ensures a systematic and transparent evaluation of the quality of the studies included in this review.

### Meta-Analysis

#### Meta-analysis on sensitivity

**Figure 2:**
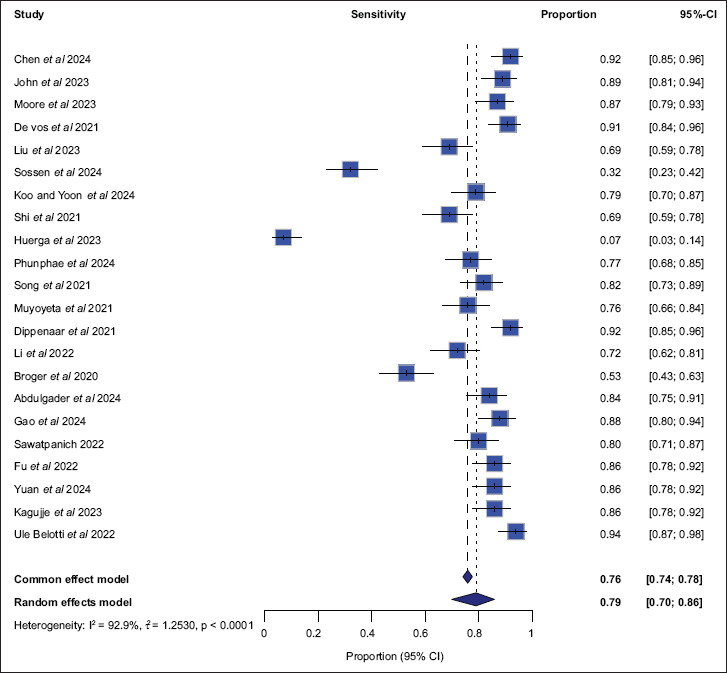
Forest plot showing the sensitivity analysis of the approaches evaluated.

The forest plot for sensitivity analysis included 22 studies, with individual sensitivity estimates ranging from 0.07 (95% confidence interval [CI]: 0.03–0.14) to 0.94 (95% CI: 0.87–0.98). Most studies reported high sensitivity, with values above 0.70, indicating strong diagnostic performance in correctly identifying true positive cases. However, notable exceptions included Huerga et al.^[25]^ (2023) with a sensitivity of 0.07 (95% CI: 0.03–0.14) and Sossen et al.^[14]^ (2024) with a sensitivity of 0.32 (95% CI: 0.23–0.42), suggesting variability in test performance across different populations or methodologies. The common effect model yielded a pooled sensitivity estimate of 0.76 (95% CI: 0.74–0.78), while the random effects model, accounting for significant heterogeneity (I^2^ = 92.9%), produced a slightly higher estimate of 0.79 (95% CI: 0.70–0.86). The high heterogeneity (p < 0.0001) [Figure 2] indicates substantial variability across studies, likely due to differences in study design, sample characteristics, or diagnostic thresholds.

#### Meta-analysis on specificity

**Figure 3:**
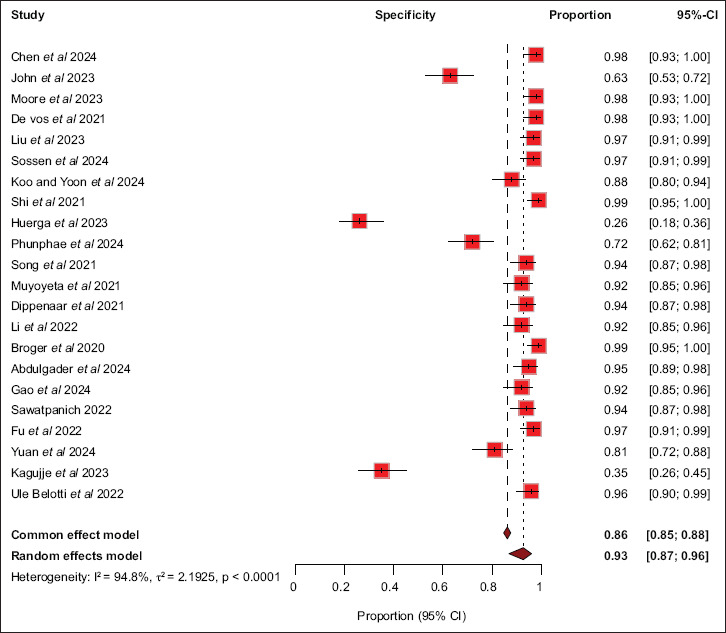
Forest plot showing the specificity of the approaches evaluated.

The specificity forest plot also included 22 studies, with estimates ranging widely from 0.26 (95% CI: 0.18–0.36) in Huerga et al.^[25]^ (2023) to 0.99 (95% CI: 0.95–1.00) in Shi et al.^[15]^ (2021) and Broger et al.^[27]^ (2020). Most studies demonstrated high specificity (>0.90), reflecting strong performance in correctly identifying true negative cases. The common effect model estimated a pooled specificity of 0.86 (95% CI: 0.85–0.88), while the random effects model, adjusted for high heterogeneity (I^2^ = 94.8%*, p*<0.0001), provided a higher estimate of 0.93 (95% CI: 0.87–0.96) [Figure 3].

#### Publication bias assessment

**Figure 4:**
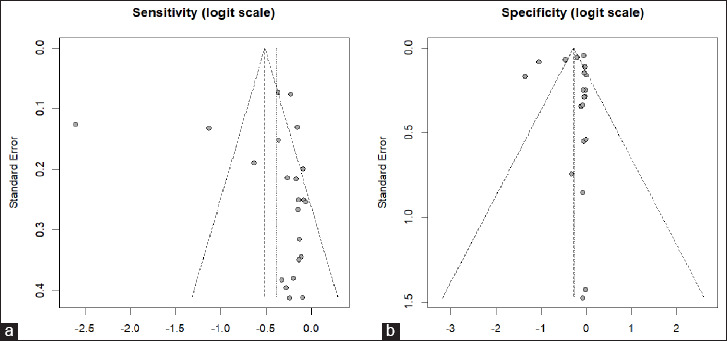
Funnel plots of diagnostic accuracy parameters: (a) sensitivity and (b) specificity on the logit scale. Each grey circle represents one study included in the meta-analysis. The dotted lines indicate the expected distribution of studies in the absence of publication bias. Symmetrical dispersion of points within the funnel suggests little or no publication bias, whereas asymmetry or clustering to one side may indicate potential publication bias.

Funnel plots were created to evaluate potential publication bias in both sensitivity and specificity analyses, using the logit scale. A visual inspection of the funnel plot for sensitivity and specificity showed a symmetrical distribution, and the Egger’s test for both sensitivity (*p* = 0.57) and specificity (*p* = 0.94). Indicating no significant evidence of publication bias [Figure 4].

### AI-Based Approach

According to previous studies retrieved from online databases, the emergence of AI and deep learning algorithms has significantly enhanced TB diagnostics.^[35,36]^ AI-powered portable chest X-ray (CXR) has proven to be an effective TB diagnostic tool^[31]^ as it is rapid with consistent image interpretation.^[37]^ A study conducted in Cambodia targeted adults with pulmonary TB and found that they are effective TB screening tools with improved performance over the conventional CXR.^[38]^ Another study on a convolutional neural network with 45 samples revealed that AI-based approaches hold a promising future for TB detection.^[39]^

### Biomarker-Based Approach

Systematic review and meta-analyses revealed that biomarker-based screening has also proven reliable for TB screening.^[40]^ One study demonstrated that the C-reactive protein (CRP) performed remarkably in screening patients with TB. It is a non-specific inflammatory marker.^[41]^ It is a valuable tool for TB screening.^[41]^ CRP testing has been added to the WHO guidelines as a point-of-care TB diagnostic tool for people living with human immunodeficiency virus (HIV) in high TB-endemic settings.^[42]^ It is rapid, cost-effective, and has a higher specificity and sensitivity of 58% and 81%, respectively, than the symptom-based TB diagnostic approach.^[42,43]^ Another set of biomarkers includes systemic inflammation response index and cardiometabolic index, which are novel biomarkers for effective multidrug-resistant TB (MDR-TB) screening.^[28]^ The integration of these two biomarkers has been effective in enhancing the screening process for MDR-TB.^[28]^

The e-nose is an innovative and user-friendly tool that utilizes an array of sensors to effectively identify and diagnose diseases by analyzing the patterns of volatile organic compounds produced by MTB. This approach enhances disease detection and contributes to improved health outcomes.^[44]^

The lateral flow urine lipoarabinomannan (LF-LAM) assay is based on the production of lipoarabinomannan (LAM) by MTB, a cell wall byproduct that can be detected in the sputum, urine, and blood of TB-infected individuals.^[26]^ The advantage of the LF-LAM is that it is cost-effective and does not require complex infrastructure; however, the uptake is very slow.^[26]^

### Molecular-Based Approach

#### Non-cartridge-based approach

TB continues to be a major cause of morbidity and mortality globally.^[1]^ Early and accurate diagnosis plays a significant role in controlling the spread of MTB. Studies retrieved from databases highlighted the performance of the non-cartridge-based molecular approach. One study evaluated the performance of TB-LAMP and revealed that this approach has positively impacted TB diagnosis due to its high sensitivity and specificity.^[45]^ Another Study found it to be instrumental in both primary and secondary healthcare settings, enabling early detection of TB.^[46]^ Systematic review and meta-analysis on TB LAMP, including 13 studies, found that this approach has so far complemented TB diagnosis in intermediate-to-high burden TB countries, especially where AFB microscopy remained the predominant approach.^[47]^ However, TB LAMP needs to be refined for better detection of TB.

#### Cartridge-based molecular approach

The studies included in this systematic review have identified various innovative diagnostic approaches for diagnosing TB. Smear microscopy is used to detect TB. However, this approach cannot differentiate between MTB and NTM infections.^[13,48]^ Molecular techniques based on cartridge-based nucleic acid amplification test (CB-NAAT) have been developed for rapid and effective TB diagnosis. Each of these approaches has shown promising results as TB diagnostic tools. Studies have evaluated GeneXpert MTB/RIF, BD MAX MTB, Meltpro, and Truenat MTB assays with patients ranging from pediatric, adult, and people living with HIV.^[13,19,49,50]^ These approaches have significantly enhanced TB diagnostics. A hospital-based study highlighted the performance of CB NAATs by being highly sensitive among people with both pulmonary and extra-pulmonary TB.^[51]^ The use of CB NAAT has remarkably enhanced TB diagnostics and has true diagnostic potential, especially among patients missed by conventional approaches. A meta-analysis of the CB-NAAT for diagnosing TB, especially in smear-negative and extrapulmonary cases, has shown promising results. CB-NAAT provides a rapid and accurate method for detecting MTB and identifying rifampicin resistance, which enhances patient management.^[52]^ Studies indicate that CB-NAAT demonstrates high specificity compared to culture methods, although its sensitivity can vary. Its value is particularly notable in cases where conventional diagnostic methods yield inconclusive results.^[52]^

#### Targeted next-generation sequencing (TNGS)

A systematic review and meta-analysis on using sequencing to diagnose TB pointed out that TNGS proved to be highly effective for the diagnosis of TB and resistance detection,^[53]^ thereby increasing the rate of TB detection from primary clinical samples.^[53]^ Another previous study that evaluated TNGS revealed that it is a sensitive, feasible, and effective TB diagnostic tool for rapid TB detection and resistance profiling (Kambli et al., 2021).^[54]^ However, its cost-effectiveness needs to be addressed, especially in low- and middle-income countries.

### Capture-XT

A study conducted at the University of London evaluated this emerging technology and revealed that it could significantly enhance TB diagnostics, thereby facilitating low-cost TB screening.^[55]^ This assay is capable of detecting resistance markers, making it suitable for both TB and drug resistance detection.^[55]^ In addition to detection, it can also quantify MTB for sequencing, which enhances downstream analysis of MTB.^[56]^ While this technology shows promise for TB detection, there is currently limited clinical data to support its effectiveness. Therefore, more studies are needed to understand its real-life applications fully.

### Study Distribution

**Figure 5:**
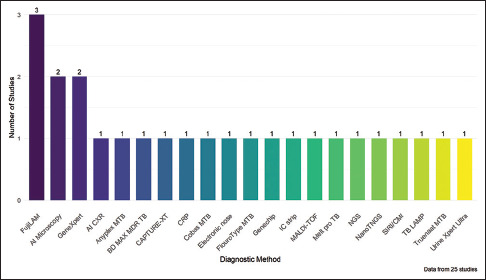
The study distribution of the diagnostic methods evaluated.

According to this review, molecular diagnostic approaches were found to be predominant among the diagnostic methods evaluated, with 13 studies, Biomarker with 5, and AI-based with 3 [Figure 5].

### Challenges Associated with TB Diagnosis

#### Limited access to diagnostics in resource-constrained settings

Many countries in TB-endemic regions lack the facilities (e.g., laboratories and trained personnel) to deploy and effectively implement advanced diagnostics, such as molecular platforms or imaging tools.^[17,29]^ Decentralized testing is underutilized due to reliance on centralized, high-throughput systems, which delay diagnosis and treatment.^[17]^

#### Complexity of new techniques

The introduction of advanced diagnostic tools, such as the GeneXpert MTB/RIF assay, has improved TB diagnosis by providing rapid and precise results. However, these technologies are challenging, particularly in settings with limited resources. A systematic review pointed out that inadequate training of healthcare workers and poor facilities hinder the effective implementation of such diagnostic tools. Diagnostic delays due to these challenges can lead to extended transmission and worsened patient outcomes.^[9]^

#### Geographical variability

There is a remarkable geographical disparity in the availability of TB diagnostic services across regions. A study evaluating the distribution of TB diagnostic equipment in Nigeria found notable disparities between urban and rural areas and between the public and private healthcare sectors. For example, GeneXpert machines were mostly available in tertiary public health centers in urban areas, with limited or no availability in primary healthcare centers, particularly in rural areas. This unequal distribution hinders timely diagnosis and treatment in underprivileged regions, contributing to ongoing transmissions.^[9]^

#### Emerging drug-resistant TB

The rise of MDR-TB requires cutting-edge diagnostic tools that can detect resistance to both first- and second-line anti-TB drugs. However, these tools are expensive and have limited availability.^[17]^ Therefore, there is a need for the deployment of sophisticated equipment for better TB detection.

### Future Direction of TB Diagnosis

Future directions for TB diagnostics focus on developing universal, high-sensitivity tools that work across diverse settings and populations, including those with paucibacillary, extrapulmonary, and pediatric TB cases. Emphasis will be placed on point-of-care testing, which is portable, affordable, and sustainable, particularly in resource-limited settings. The emergence of innovative techniques will pave the way for broad TB control, and portable devices such as electronic noses will greatly impact community TB screening. In most LMICs, health-seeking behavior is very low; therefore, the development of such innovative, sensitive, and portable diagnostic tools will contribute immensely to TB control.

Biomarker-based test integration, improved drug resistance detection, and leveraging advances in AI will promote diagnostic accuracy and efficiency.

Exploring other sensitive, rapid, cost-effective, and sustainable diagnostic techniques may be an option. Collaboration between academia, industry, and policymakers will be vital to ensure the rapid translation of these novel diagnostics into real-world applications. The goal is to create flexible, sustainable, and eco-friendly innovations that support the prevention, early detection, and treatment monitoring for the reduction of TB transmission and improve outcomes across the globe.

## DISCUSSION

The burden of TB remains a public health burden especially in low-resources settings where low diagnostic infrastructure but with populations at risk for TB including MCH populations and individuals with HIV/AIDS. Our review found innovative approaches for TB diagnosis, with improved sensi-tivityandspecificityrangingfrommolecular,biomarker-based, and 0.70–0.86, I^2^=94.8%, p<0.0001 and a pooled specificity of AI-based approaches pooled sensitivity of 0.79 (95% CI: 0.93 (95% CI: 0.87–0.96, I^2^=94.8%, p<0.0001). This is similar to the studies conducted on Molecular and AI-based approaches.^[57-61]^ However, our findings are slightly higher than the studies conducted on LAM in People living with HIV, with a pooled sensitivity of 42% and a pooled specificity of 91%.^[62]^ Previous studies might have focused on specific approaches or other parts of TB diagnosis^[63]^ however, this review takes a broader approach by covering recent trends in the TB diagnostic landscape to ensure that TB is properly managed. Ultimately, our review is unparalleled by incorporating sequencing and artificial intelligence to diagnose TB^[20,64]^ and not only integrating these techniques but also comparing their diagnostic accuracy with that of current diagnostic approaches from previous studies, thereby offering a clear perspective on TB diagnosis. This approach supports the findings of Bartolomeu-Gonçalves et al.^[65]^ In terms of methodological rigor, our systematic review employed a stringent approach, adhering to predefined inclusion and exclusion criteria, including studies published within the last five years.

Additionally, our review stands out from previous studies by more broadly addressing the challenges associated with TB diagnosis.^[63]^ Our review adapts qualitative and quantitative syntheses to provide a comprehensive picture of TB diagnosis by discussing the approaches broadly, highlighting their diagnostic accuracy, potential, and implications, their pooled sensitivity and specificity, as well as the associated challenges. This approach is critically valuable for clinicians and policymakers willing to make evidence-based decisions regarding the adoption of novel diagnostic approaches. However, exploring innovative approaches and advanced diagnostic tools, such as AI-based, biomarker-based, and sequencing technologies, might be challenging. These studies reported that cost, special expertise requirements, and limited laboratory infrastructure are common implementation barriers. Therefore, further studies should focus on the feasibility and cost-effectiveness of these approaches to inform policymakers.

Molecular methods based on NAATs have been developed to address the setbacks of conventional approaches. NAAT systems are rapid and easy to operate, increasing the chances of early detection and treatment.^[2,66]^ Studies conducted on NAATs among suspected patients with EPTB in Philippines and patients with HIV in US revealed that NAATs are a quick and effective TB diagnostic tool.^[67,68]^

Another set of molecular diagnostic approaches includes cartridge-based NAATs. Cartridge-based nucleic acid amplification tests (CB-NAATs) are mostly automated techniques that rapidly and simultaneously detect MTB and rifampicin resistance.^[6,12,17,19,22,23,50,69-71]^ Thesestudiesevaluated CB-NAATS among patients with EPTB and pediatric patients; their finding suggests that it is a promising tool for TB detection.^[72,73]^

With advances in molecular biology, TNGS of MTB has also been reported to be an important tool for the detection of tuberculosis and resistance to TB drugs, thereby increasing the chance for rapid TB detection and treatment monitoring.^[20,74-78]^ Wu et al evaluated the accuracy of TNGS in TB detection using non-sputum specimen and found it to be effective for both TB and resistance detection.^[79]^

Several biomarker-based diagnostic methods have been developed and evaluated to end TB. These approaches have shown promising sensitivity and could be used as point-of-care tests for TB.^[28,29,80-82]^

A prominent study on artificial intelligence for TB detection was conducted by John et al.^[31]^ The researchers evaluated computer-aided chest radiography (CA-CXR) using the qXR version 3 software. The study was conducted in Nigeria with a sample size of 5297, and the researchers found that qXR is an effective tool for active TB screening and could greatly enhance TB diagnosis globally.^[31]^ Although this study was conducted in one of the TB-endemic countries, a multicenter study with a larger sample size is needed to fully understand the accuracy of this new technology. In another study on CAPTURE-XT, a sustainable and rapid TB diagnostic tool, which was conducted by Moore et al.^[11]^ using 100 biobanked specimens, the technique was proven to be an effective TB diagnostic tool with considerable specificity and sensitivity; however, more research using multicenter and larger sample sizes is needed to fully assess the accuracy of this innovative technique.

### LIMITATIONS

#### Narrowed cost-effectiveness review

This systematic review highlighted several innovative techniques, but could not provide a cost analysis of these techniques; therefore, only their diagnostic accuracy was highlighted. Moreover, only a few studies have mentioned cost-effectiveness and accessibility, particularly in settings with limited resources.

##### Language Bias

In our study selection process, we included only articles published in English. This decision was made due to resource constraints and our lack of proficiency in other languages. However, it may have introduced systematic bias. Important studies published in different languages could have been unintentionally excluded, potentially affecting the comprehensiveness of our evidence base. This exclusion might lead to an overestimation or underestimation of the diagnostic accuracy of the tools we reported. Consequently, restricting our literature review to English-only sources may have skewed our findings in favor of more positive outcomes, thereby impacting both the validity and generalizability of our results.

##### Potential Diagnostic Error

A key limitation of this review is the potential for diagnostic errors in the primary studies. Misclassifications, whether false positives or false negatives, can significantly affect the accuracy measures (sensitivity and specificity) reported. These errors may result from suboptimal reference standards, operator-dependent techniques, variations in sample quality, or inconsistent diagnostic protocols. Some studies used imperfect or non-uniform reference standards, introducing verification bias that impacts the estimation of true diagnostic performance. In addition, variations in laboratory conditions and personnel expertise can lead to intra- and inter-observer variability, further compounding inaccuracies. These diagnostic errors threaten the internal validity of individual studies and can lead to heterogeneity in pooled estimates, weakening conclusions from meta-analysis. Although we used statistical models to address heterogeneity, we acknowledge that diagnostic inaccuracies may still distort the overall findings.

##### Gaps in Pediatric and EPTB

A significant limitation of this review is the lack of studies focused on pediatric TB and EPTB. Most diagnostic evaluations were conducted in adults with pulmonary TB, limiting the applicability of our findings to children and EPTB patients. Diagnosing TB in children is challenging due to non-specific symptoms, difficulties in obtaining sputum samples, and a lower bacterial load. EPTB, affecting areas other than the lungs, often requires invasive specimen collection and may not be detected by tests designed for pulmonary samples. Consequently, the sensitivity and specificity observed in adult pulmonary TB cases might not reflect the true performance of these tools in children and EPTB patients, potentially leading to underdiagnosis or delayed diagnosis. Future studies should include diverse populations, especially pediatric and EPTB patients, to provide more equitable evidence on diagnostic tool performance across all TB forms.

## CONCLUSION AND GLOBAL HEALTH IMPLICATIONS

TB remains a significant global health challenge, necessitating the development of a range of innovative approaches to enhance diagnostic efficiency. These advancements in diagnostic techniques offer improved accuracy and reduced turnaround times compared to conventional methods. This systematic review highlights the transformative potential of progressive diagnostic strategies for TB detection, including molecular, biomarker-based, and AI-driven methods. These innovations not only enhance diagnostic precision but also expand access to diagnostics, particularly in low-resource settings where the burden of TB is notably high. Nevertheless, the successful implementation of these innovative diagnostic approaches poses considerable challenges, especially in regions with limited resources. Governments must create strategic plans that facilitate the integration of such cutting-edge technologies into national TB management frameworks. In addition, targeted investments in context-appropriate technologies, the formulation of equity-focused policies, and the pursuit of rigorous research will be vital to achieving global TB elimination targets by the year 2030.

## COMPLIANCE WITH ETHICAL STANDARDS

### Conflicts of Interest

*Dr. Hamisu Salihu is the Executive* **Editor of the journal. He did not participate in making editorial decisions for this article.**

### Financial Disclosure

Nothing to declare.

### Ethics Approval

Not applicable.

### Declaration of Patient Consent

**Patient’s** consent not required as there are no patients in this study.

### Acknowledgments

None.

### Use of Artificial Intelligence (AI)-Assisted Technology for Manuscript Preparation

The authors confirm that there was no use of artificial intelligence (AI)-assisted technology for assisting in the writing or editing of the manuscript, and no images were manipulated using AI.

### Disclaimer

None.

#### Key Messages

1. This review has identified that several innovative methodologies have been developed for the diagnosis of tuberculosis (TB). 2. Cartridge-based molecular tests, such as GeneXpert and Truenat, have demonstrated strong efficacy, especially in cases of smear-negative and extrapulmonary TB. 3. Artificial intelligence–based diagnostic solutions and biomarker-driven approaches have also shown significant promise for enabling rapid and effective TB screening. 4. Despite these advancements, limited access, geographical disparities, and the rise of multidrug-resistant (MDR) tuberculosis continue to hinder global diagnostic progress. 5. To address these challenges, policymakers, governments, and key stakeholders should formulate a strategic plan that ensures broad and equitable diagnostic coverage. 6. Such efforts must align with the World Health Organization’s (WHO) 2030 mission to enhance TB control and ultimately reduce disease burden worldwide.
